# Migraine and risk of hemorrhagic stroke: a study based on data from general practice

**DOI:** 10.1186/1129-2377-15-74

**Published:** 2014-11-11

**Authors:** David Gaist, Antonio González-Pérez, Messoud Ashina, Luis Alberto García Rodríguez

**Affiliations:** 1 Department of Neurology, Odense University Hospital and Institute of Clinical Research, Faculty of Health Sciences, University of Southern Denmark, Sdr Boulevard 29, 5000 Odense, Denmark; 2 Andalusian Bioinformatics Research Center (CAEBi), Seville, Spain; 3 Department of Neurology & the Danish Headache Center, Glostrup University Hospital and Faculty of Health and Medical Sciences, University of Copenhagen, Copenhagen, Denmark; 4 Spanish Centre for Pharmacoepidemiologic Research (CEIFE), Madrid, Spain

**Keywords:** Intracerebral hemorrhage, Subarachnoidal hemorrhage, Risk factors, Migraine

## Abstract

**Background:**

We investigated the association between hemorrhagic stroke and migraine using data from The Health Improvement Network database.

**Findings:**

We ascertained 1,797 incident cases of intracerebral hemorrhage (ICH) and 1,340 of subarachnoid hemorrhage (SAH). Density-based sampling was used to select 10,000 controls free from hemorrhagic stroke. Using unconditional logistic regression models, we calculated the risk of hemorrhagic stroke associated with migraine, adjusting for age, sex, calendar year, alcohol, body mass index, hypertension, previous cerebrovascular disease, oral contraceptive use, and health services utilization.

The risk (odds ratio [OR]) of ICH among migraineurs was 1.2 (95% confidence interval [CI] 0.9–1.5), and of SAH was (1.2, 95% CI 0.9–1.5). The association with ICH was stronger for migraine diagnosed ≥20 years prior to ICH (OR 1.6, 95% CI 1.0–2.4), but not with SAH (OR 1.1, 95% CI 0.6–2.1). In analyses stratified by migraine type and gender, the OR of ICH in women with migraine with aura was 1.7 (95% CI 0.9–3.4) and the corresponding OR of SAH in women was 1.2 (95% CI 0.6–2.3).

**Conclusion:**

No clear increased risk of ICH or SAH was observed in migraineurs.

## Introduction

Migraine is a common disorder [1]. A potential link between migraine and vascular disease is therefore of great interest from a scientific and a public health perspective. A recent meta-analysis, based on 8 epidemiological studies, concluded that migraine may increase the risk of hemorrhagic stroke [2]. However, the results did not allow conclusions to be drawn on hemorrhagic stroke type [2]. We investigated the relationship between migraine and hemorrhagic stroke subtypes in the general population, using data from The Health Improvement Network (THIN) database [3,4].

## Methods

The collection of data into THIN was approved by the South-East Multicentre Research Ethics Committee in the United Kingdom. The study methodology has been described in detail in previous publications [3,4]. In brief, the study cohort comprised all individuals within the UK medical research database THIN aged 20 to 89 years between January 2000 and December 2008. Individuals with a diagnosis of ICH or SAH before their study start date were excluded from the study cohort. Members in the study cohort were followed from the start date until the earliest occurrence of one of the following endpoints: a) hemorrhagic stroke recorded, b) the patient reached 90 years of age, c) patient death, or d) the end of the study period (December 2008). For all patients identified with codes compatible with hemorrhagic stroke, free text comments were requested and the computerized patient profiles were manually reviewed. Cases were discarded during this review in the following instances: the initial diagnostic suspicions were not confirmed, the event was ischemic rather than hemorrhagic, the case was prevalent rather than incident (i.e., not a primary event), the stroke was secondary to trauma, or the patient experienced intracranial extracerebral hemorrhage other than SAH (e.g., subdural hematoma). Those not discarded after manual review were retained as cases. A validation study, based on additional data collected from primary care physicians and described in detail elsewhere, confirmed that 84% of cases thus identified fulfilled the criteria for the study [3].

### Nested case–control analysis

To evaluate the risk of hemorrhagic stroke associated with migraine, we performed a case–control study nested in the study cohort. In this analysis, we included as cases all hemorrhagic strokes, using the date of diagnosis as the index date. A group of 10,000 controls within THIN was randomly selected from the pool of eligible person-time (i.e., using density-based sampling with no replacement) and was frequency-matched to all cases for sex, age (±1 year), and calendar year of diagnosis. For all cases and controls, we ascertained demographic (e.g., age, sex, body mass index) and lifestyle (e.g., smoking, alcohol intake) factors, as well as general comorbidities at the index date; the distribution of these factors among cases and controls has been previously presented [4].

### Migraine diagnosis prior to index date

We ascertained whether subjects suffered from migraine. Free-text comments on all subjects with Read codes compatible with a diagnosis of migraine at any time prior to the index date were requested. Computerized patient profiles were reviewed by a neurologist with a special interest in migraine (D Gaist), who was blinded with regard to case status, i.e. presence of hemorrhagic stroke. Blinding was achieved by removing records 1 month prior and after the index date for both cases and controls; furthermore, for cases, all comments that included codes for haemorrhagic stroke were removed. A diagnosis of migraine was accepted, unless free text comments contradicted it, which was the case in 8 women (classified as non-migraineurs). Subjects with verified migraine diagnosis were subclassified by migraine type, i.e. migraine with aura and migraine without aura. Only few subjects received codes compatible with migraine with aura (1 case and 10 controls). A diagnosis of migraine with aura was thus mainly reached by reviewing the clinical information included in free text comments. For patients with migraine, we calculated the time period between the first diagnosis of migraine and the index date and classified it as follows: <2, 2–9, 10–19, and ≥20 years.

### Statistical analyses

Unconditional regression analyses were run for hemorrhagic stroke and separately for ICH and SAH to estimate odds ratios (ORs), together with 95% confidence intervals (CIs). ORs presented are compared with not having migraine. The fully adjusted model included frequency-matched variables (i.e., age, sex, and calendar year) and the following covariates: smoking, alcohol, body mass index, hypertension, previous cerebrovascular disease (ischaemic stroke or transient ischaemic attack), oral contraceptive use, and health services utilization (PCP visits, referrals, and hospitalizations). We also performed analyses stratified by gender and age (≤, >60 years). All analyses were performed using Stata SE 10.0 (StataCorp, College Station, TX).

## Findings

A history of migraine was present in 5.5% subjects with ICH, 8.1% subjects with SAH, and 5.2% controls (Table 1). Migraine was associated with a non-significantly increased risk of hemorrhagic stroke (odds ratio 1.2, 95% CI 1.0-1.4; P value .078), ICH (OR 1.2, 95% confidence interval [CI] 0.9–1.5), and SAH (OR 1.2, 95% CI 0.9–1.5). In analyses by subtype of migraine the association remained unchanged. However, the risk estimate of SAH was not increased in women with migraine with aura (OR 0.9, 95% CI 0.5-1.7) (Table 1). Analyses of duration since first recorded migraine diagnosis revealed that, for migraine, the association was only present among subjects with a first diagnosis of this headache ≥20 years prior to ICH (OR 1.6, 95% CI 1.0–2.4) (*P* value .05), while for SAH no clear pattern was found (Table 1). The association between migraine and ICH was most pronounced for subjects with migraine with aura aged <60 years at the time of their hemorrhagic stroke (OR 1.8, 95% CI 0.7-4.4). Male gender was not associated with increased risk of ICH or SAH (Table 1). Women, however, were at increased risk of ICH, particularly if they suffered from migraine with aura (OR 1.7, 95% CI 0.9–3.4); the risk of SAH in women with migraine with aura was only slightly raised compared with non-migraineurs (OR 1.2, 95% CI 0.6–2.3). With the exception of duration of migraine history (borderline significant), none of the above mentioned associations were statistically significant.

**Table 1:** Migraine and risk for intracranial hemorrhage (ICH) and subarachnoidal hemorrhage (SAH)

	**Number**				**Adjusted OR (95% CI)**^**a**^		
	**ICH**	**SAH**	**Overall hemorrhagic stroke**	**Controls**	**ICH**	**SAH**	**Overall hemorrhagic stroke**
No migraine	1,698	1,231	2,929	9,476	1 (reference)	1 (reference)	1 (reference)
Migraine	99	109	208	524	1.2 (0.9-1.5)	1.2 (0.9-1.5)	1.2 (1.0-1.4)
Migraine without aura	84	97	181	450	1.2 (0.9-1.5)	1.2 (1.0-1-6)	1.2 (1.0-1.4)
Migraine with aura	15	12	27	74	1.2 (0.7-2.2)	0.9 (0.5-1.7)	1.0 (0.7-1.6)
Migraine duration^b^, years							
<2	15	17	32	85	1.1 (0.6-1.9)	1.0 (0.6-1.7)	1.0 (0.7-1.6)
2-9	35	55	90	213	1.1 (0.8-1.6)	1.3 (1.0-1.8)	1.2 (0.9-1.6)
10-19	22	25	47	134	1.1 (0.7-1.8)	1.1 (0.7-1.7)	1.1 (0.8-1.6)
≥20	27	12	39	92	1.6 (1.0-2.4)^c^	1.1 (0.6-2.1)	1.3 (0.9-2.0)
Age <60 years^d^							
Migraine without aura	25	69	94	202	1.2 (0.7-1.9)	1.3 (1.0-1.8)	1.2 (0.9-1.6)
Migraine with aura	6	12	18	36	1.8 (0.7-4.4)	1.3 (0.7-2.6)	1.4 (0.8-2.6)
Age ≥60 years^d^							
Migraine without aura	59	28	87	248	1.2 (0.9-1.6)	1.1 (0.7-1.6)	1.1 (0.9-1.4)
Migraine with aura	9	0	9	38	1.0 (0.5-2.1)	NA^e^	0.7 (0.3-1.4)
Men							
Migraine without aura	28	19	47	127	1.2 (0.8-1.9)	1.1 (0.7-1.9)	1.1 (0.8-1.6)
Migraine with aura	4	0	4	27	0.7 (0.2-2.3)	NA^e^	0.4 (0.2-1.3)
Women							
Migraine without aura	56	78	134	323	1.2 (0.9-1.6)	1.3 (1.0-1.7)	1.2 (1.0-1.5)
Migraine with aura	11	12	23	47	1.7 (0.9-3.4)	1.2 (0.6-2.3)	1.4 (0.8-2.3)

^a^Adjusted for sex, age, calendar year, smoking, alcohol, body mass index, hypertension, previous cerebrovascular disease, oral contraceptive use, and health services utilization.

^b^Time from first recorded migraine diagnosis to index date.

^c^p value ≤ .05.

^d^At index date.

^e^Not applicable.

## Discussion

The major outcome of this study, based on an analysis of data from more than 3,000 patients with hemorrhagic stroke is that it does not provide evidence of a clear increased risk of ICH or SAH in sufferers of migraine. Although relative risk estimates were slightly to moderately increased for certain subgroups, i.e., the increased risk of ICH in migraineurs with a long history of migraine, and in women and subjects under age 60 with migraine with aura, the results did not reach statistical significance.

A recent meta-analysis reported an overall pooled adjusted risk estimate (RR) of hemorrhagic stroke of 1.48 (95% CI 1.16-1.88), which was slightly higher in subjects with migraine with aura (RR 1.62, 95% CI 0.87-3.03) [2]. Although of a smaller magnitude, the relative risk estimates in our study are similar regarding the association of ICH with migraine, particularly regarding migraine with aura in women. We found that the risk of ICH increased in subjects with a long history of migraine, albeit not statistically significantly. A prospective study, based on data from the Women’s Health Study reported increased rates of hemorrhagic stroke for women with migraine towards later years of follow-up, but the risk increase was only observed in women with active migraine with aura at baseline [5]. In our study, we lacked information that would enable reliable identification of women with active migraine with aura, a factor that may have contributed to the lower risk estimates found in our study compared with Kurth et al.

Although the meta-analysis did not indicate that risk of hemorrhagic stroke was modified by age, we note that our findings of higher risk estimates in younger subjects are in line with those of a recently published large study [6]. Finally, our finding of a low risk of SAH associated with migraine is in agreement with the largest of two studies that addressed this issue [5,7].

Our study strengths include reliance on a general practice based registry, THIN, enabling prospective long-term follow-up of a large general population in the UK with continuous registration of health-related events and information on several potential confounders. The validity of the hemorrhagic stroke diagnosis in this setting is high [3].

Our study also has a number of potential limitations. In spite of the large number of cases of haemorrhagic stroke, there were few cases with antecedents of migraine with aura. This limited the statistical power of these analyses. Furthermore, the diagnosis of migraine was not established according to the International Headache Society criteria [8]. The migraine diagnosis was, however, reached by general practitioners (GPs) and as such reflects everyday practice. Not all migraineurs seek medical attention for their migraine headaches [9]. We find it probable that migraineurs with milder attacks were less likely to seek medical attention and may therefore be underrepresented in our study, which may explain our finding of a rather low prevalence of migraine. We sub-classified migraine type almost entirely based on free-text comments, since GPs mostly did not use codes for migraine with aura, even in cases where they clearly stated this diagnosis and described aura symptoms in the free-text. This probably means that some patients with migraine with aura in our sample have been misclassified as migraine without aura, due to missing information on aura symptoms in the free-text. We believe that this source of non-differential misclassification probably leads to an underestimate of the association of migraine with aura and hemorrhagic stroke. We therefore conclude that our study indicates that migraine, and in particular migraine with aura, may be associated with an increased risk of ICH, but probably not SAH. However, given the implications of a link between migraine and ICH, and the limitations of the present as well as previous studies, the issue merits further studies.

## Competing interests

The original data collection and study was supported by a research grant from Bayer Pharma AG to the Spanish Centre for Pharmacoepidemiologic Research (CEIFE).

## Authors’ contribution

DG contributed to the study concept and design, the analysis and interpretation of data and the acquisition of data. AG-P contributed to the acquisition of data and the statistical analysis of data and the interpretation of data. LAGR contributed to the study concept and design, the analysis and interpretation of data and the acquisition of data. MA contributed to the analysis and interpretation of data. All authors contributed to the drafting/revising of the manuscript for content, and read and approved the final manuscript.
